# HOXA13 promotes the proliferation, migration, and invasion of nasopharyngeal carcinoma HNE1 cells by upregulating the expression of Snail and MMP-2

**DOI:** 10.1038/s41598-023-40041-8

**Published:** 2023-08-10

**Authors:** Jinping Liu, Huajun Feng, Dingting Wang, Yuanyuan Wang, Jian Luo, Shengen Xu, Feipeng Zhao, Gang Qin

**Affiliations:** 1 Department of Otolaryngology Head and Neck Surgery, The Affiliated Hospital of Southwest Medical University, NO: 25, Taiping Street, Jiangyang District, Luzhou, 646000 China; 2 Department of Otolaryngology Head and Neck Surgery, The First People’s Hospital of Yibin, Yibin, 644000 China

**Keywords:** Cancer, Molecular biology, Biomarkers, Diseases

## Abstract

Homeobox A13 (HOXA13) has been verified as an oncogen in some malignancies. However, its role in nasopharyngeal carcinoma (NPC) is still unclear. This study aims to explore the role of HOXA13 in NPC and its underlying mechanism. The mRNA expression of HOXA13 in NPC was obtained from the GSE53819 and GSE64634 datasets in the Gene Expression Omnibus (GEO) database. MTT, colony formation and transwell assays and xenograft tumour models were used to investigate the effects of HOXA13 on NPC HNE1 cells in vitro and in vivo. The expression of HOXA13, epithelial-mesenchymal transition-transcription factor (EMT-TF) Snail and matrix metalloproteinase 2 (MMP-2) was detected by immunohistochemistry, quantitative real-time polymerase chain reaction (qRT-PCR) and Western blotting. The results showed that HOXA13 was upregulated in NPC. Silencing HOXA13 suppressed the proliferation, migration, and invasion of HNE1 cells, which inhibited tumour growth, while overexpression of HOXA13 induced the opposite effects. In addition, the expression of Snail and MMP-2 at the transcriptional and protein levels was associated with the expression of HOXA13. In summary, our results suggest that HOXA13 plays a role as a cancer-promoting gene in NPC. The underlying mechanism may be related to the upregulation of Snail and MMP-2.

## Introduction

Nasopharyngeal carcinoma (NPC) is a malignant tumour originating from the nasopharyngeal epithelium and has the highest incidence among head and neck tumours. There were approximately 129,000 new cases of NPC in the world in 2018, and 70% of these cases were in East Asia and Southeast Asia^1^. The extremely unbalanced geographical distribution indicates that environmental carcinogenic factors play an important role in the development of NPC^2^. Epstein-Barr virus infection is a recognized risk factor for NPC; More than 98% of NPC cases are associated with latent Epstein-Barr virus infection^3,4^. Radiotherapy or combined radiotherapy and chemotherapy are the main treatment methods for NPC, and although great advancements in radiotherapy technology have reduced the mortality rate of NPC in recent years^1,5^, early cervical lymph node metastasis and distant metastasis often lead to a poor prognosis^6^. Approximately 3/4 of patients will have cervical lymph node metastasis^5^. Therefore, focusing on the molecular mechanisms underlying the proliferation, invasion, and metastasis of NPC may lead to innovative diagnostic and treatment strategies.

HOX genes are a highly conserved subgroup of genes in the homeobox superfamily. In the human genome, there are a total of 39 HOX genes in four clusters (A-D), and each gene is located on a different chromosome. These genes are transcription factors that regulate many processes, such as cell differentiation, proliferation, apoptosis, receptor signal transduction, and angiogenesis, and play vital roles in embryonic development and the occurrence and progression of cancers^7^. A growing number of studies have confirmed the abnormal expression of HOX genes in a variety of solid tumours^8–11^. HOXA13, a member of the HOX gene family, has been found to function as a cancer-promoting gene in some cancers^12–14^. HOXA13 can positively regulate the FAK/Src axis mediated by FN1 and transactivate ACLY and IGF1R to promote cancer progression^15,16^. Previously, we found that HOXA13 could regulate in the proliferation, migration and invasion of NPC as a downstream target of the lncRNA HOTTIP. Low expression of HOXA13 inhibits the EMT process and enhances the sensitivity of esophageal squamous cell carcinoma sensitizes to cisplatin by reducing Snail expression and increasing E-cadherin expression^17^. High expression of HOXA13 is often associated with a poor prognosis and shorter survival time^18^. In hepatocellular carcinoma, the expression of HOXA13 protein is elevated, and in poorly differentiated and highly malignant hepatocellular carcinoma, the expression of HOXA13 protein is further increased, and the overall survival of the patients is decreased^19^. The mechanism of action of HOXA13 in NPC needs to be further studied.

Epithelial-mesenchymal transition (EMT) is a process in which epithelial-mesenchymal transition-transcription factors (EMT-TFs) induce epithelial cells to transform into mesenchymal cells^20^. The activation of EMT leads to loss of polarity of epithelial cells, destruction of intercellular junctions, degradation of the basement membrane, and extracellular matrix (ECM) remodelling, resulting in characteristics of mesenchymal cells, and this process is considered an important mechanism of cancer progression^21,22^. Snail, a major EMT-TF^23^, is activated in the early stage of EMT and can promote EMT by inhibiting the expression of the epithelial adhesion molecule E-cadherin. Additionally, Snail can cooperate with other transcription factors, such as ZEB1, to inhibit the expression of the Crumbs cell polarity component LGL2 at the transcription level and inhibit the expression of the Pals1-related tight junction protein PATJ, which changes cell polarity, decreases or eliminates intercellular adhesion, and promotes tumour invasion and metastasis^24,25^.

Basement membrane degradation and ECM remodelling are important steps in the EMT process and are also necessary conditions for epithelial cell dispersion and mesenchymal cell invasion. Matrix metalloproteinases (MMPs), key enzymes in ECM protein degradation, play important roles in the degradation of the basement membrane, fibronectin, and collagen^26^. For example, MMP-3 can degrade a variety of ECM proteins and is regulated by HGF in hepatocellular carcinoma to maintain EMT, which promotes invasion^27^. Interestingly, there is an interaction between Snail and MMPs, wherein Snail promotes invasion in hepatocellular carcinoma by indirectly upregulating the expression of MMP-1, MMP-2, and MMP-7^28^. On the other hand, MMP-3 can stimulate the expression of Rac1b to increase the levels of reactive oxygen species in cells, promote an increase in Snail expression, and induce the EMT process^29^. However, whether HOXA13 exert a cancer-promoting effect in NPC through Snail or MMPs has not been reported.

To explore the role of HOXA13 in NPC, we analyzed the expression of HOXA13 in NPC in the Gene Expression Omnibus (GEO) database. Lentiviral transfection was used to construct HNE1 cells with stable silencing or overexpression of the HOXA13 gene, and then the effect of HOXA13 dysregulation on HNE1 cells and its potential mechanism of action were investigated in vitro and in vivo.

## Results

### HOXA13 is highly expressed in NPC

**Figure 1:**
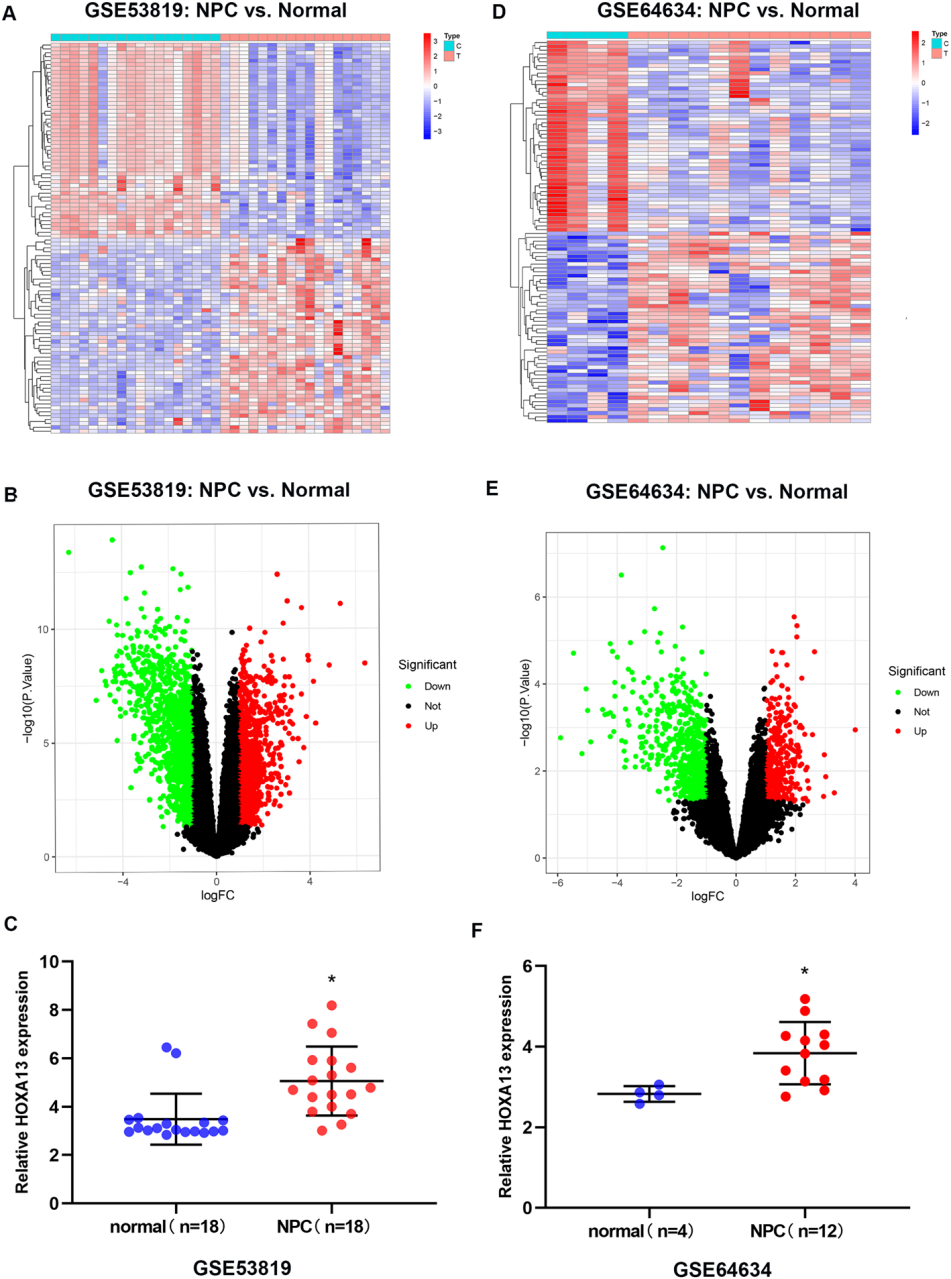
HOXA13 expression was upregulated in NPC. (**A**, **B**) Differentially expressed genes in NPC from the GSE53819. (**C**) Expression of HOXA13 in NPC tissues and normal nasopharyngeal tissues in the GSE53819. (**D**–**E**) Differentially expressed genes in NPC from the GSE64634. (**F**) Expression of HOXA13 in NPC tissues and normal nasopharyngeal tissues in the GSE64634 (*, *P* < 0.05).

**Figure 2:**
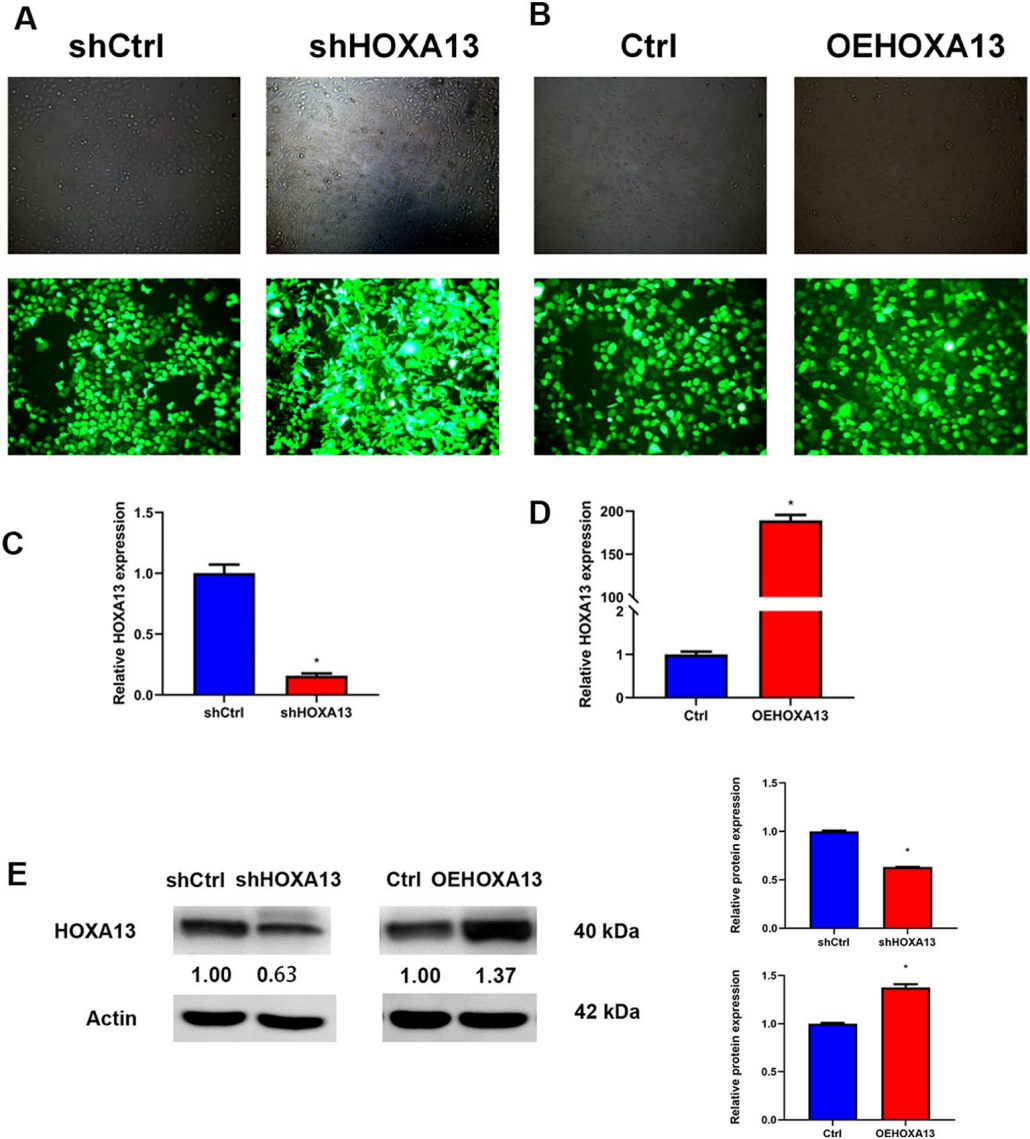
Cell transfection and validation. (**A**, **B**) Fluorescence microscopy was used to observe the transfection efficiency. (**C**, **D**) qRT-PCR assay detect HOXA13 mRNA expression in HNE1 cells after transfection. (**E**) Western blotting detect HOXA13 protein expression in HNE1 cells after transfection, the samples are derived from the same experiment and that blots were processed in parallel. Numbers represent ratios of gray value of target band to gray value of Actin. (*, *P* < 0.05).

In both GSE53819 and GSE64634, the expression level of HOXA13 in NPC tissues was higher than that in normal tissues (*P* < 0.001, *P* < 0.001) (Fig. 1A–F). To study the effect of HOXA13 on the biological characteristics of NPC cells, we constructed HOXA13-silenced and HOXA13-overexpressing cell lines using HNE1 cells. the transfection efficiency of cells was observed under a fluorescence microscope (Fig. 2A, B), and then verified by qRT-PCR and Western blotting (Fig. 2C–E). The relative mRNA and protein expression levels of HOXA13 in the shHOXA13 group were significantly lower than those in the shCtrl group (*P* < 0.05), and those in the OEHOXA13 group were significantly higher than those in the Ctrl group (*P* < 0.05).

### Silencing of HOXA13 inhibit the proliferation, migration, and invasion of NPC cells

**Figure 3:**
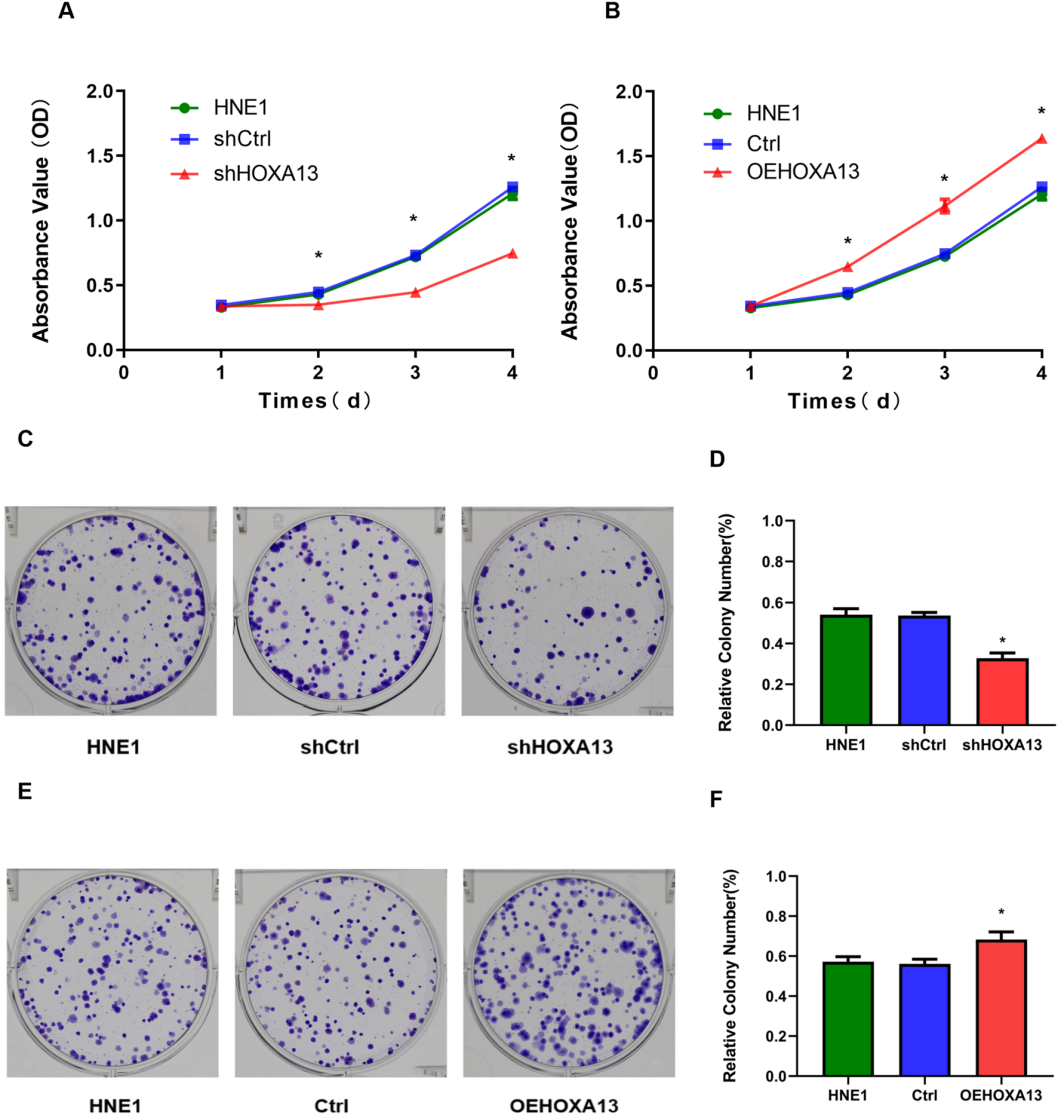
Silencing of HOXA13 inhibits NPC cell proliferation. (**A**, **B**) MTT assay. OD values of cells in each group at different time points. (**C**–**F**) Plate colony assay. Colony formation rate of HNE1 cells in each group after 7–14 days of culture. (*, *P* < 0.05).

**Figure 4:**
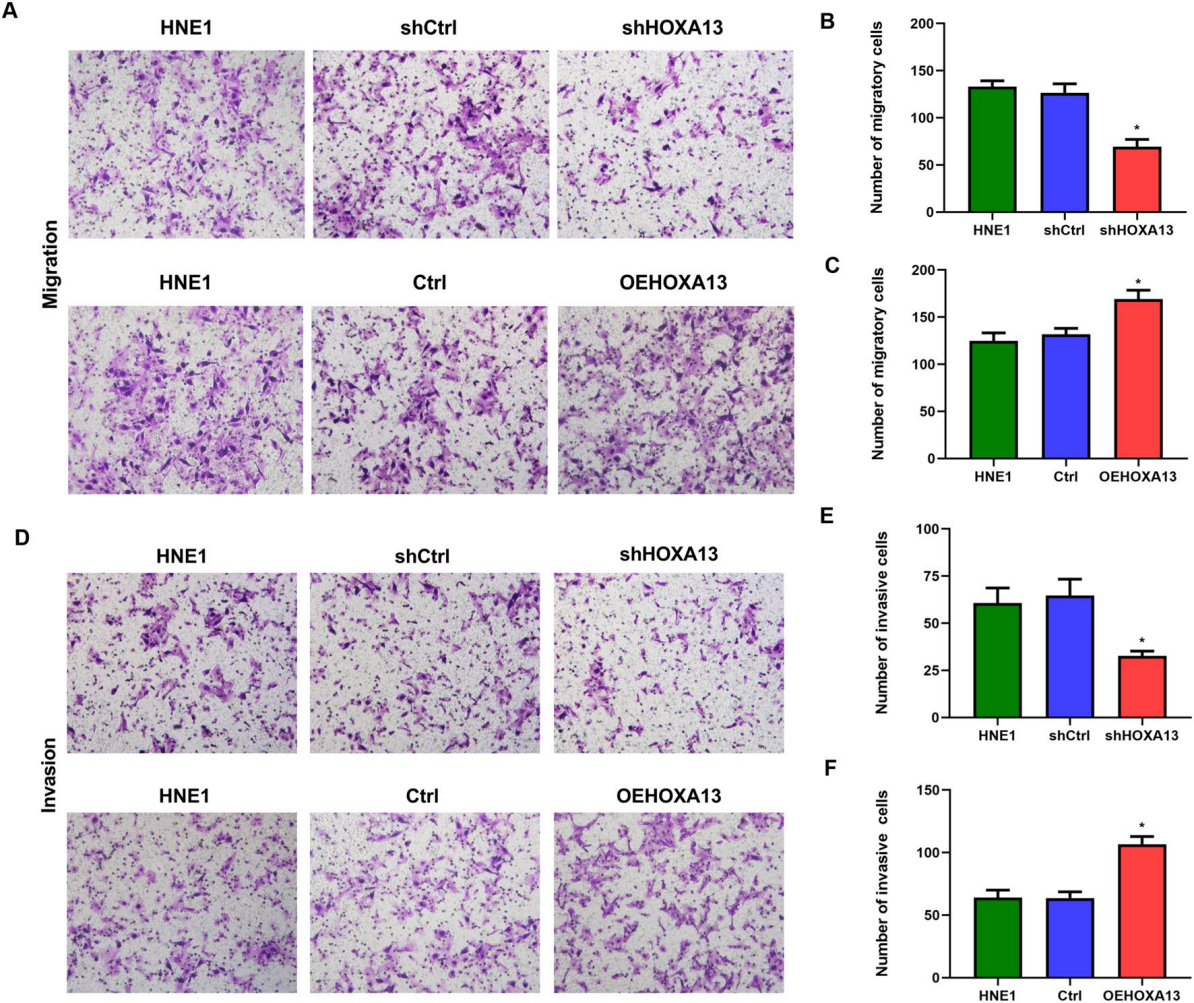
Silencing of HOXA13 inhibits migration and invasion in NPC cells. (**A**–**C**) Transwell assay. The number of cells that penetrated the membrane in each group was measured without Matrigel. (**D**–**F**) The number of cells that penetrated the membrane in each group was measured with Matrigel. (*, *P* < 0.05).

Through MTT and plate colony formation assays, we found that the inhibition of HOXA13 significantly decreased the viability and proliferation of HNE1 cells, while the overexpression of HOXA13 significantly increased the viability and proliferation of HNE1 cells (Fig. 3A–F). In addition, transwell assays showed weakened migratory ability in HNE1 cells transfected with shHOXA13 and enhanced migratory ability in HNE1 cells transfected with OEHXOA13 compared with control cells (Fig. 4A–F).

### Silencing of HOXA13 inhibit the growth of HNE1 cell xenograft tumours

**Figure 5:**
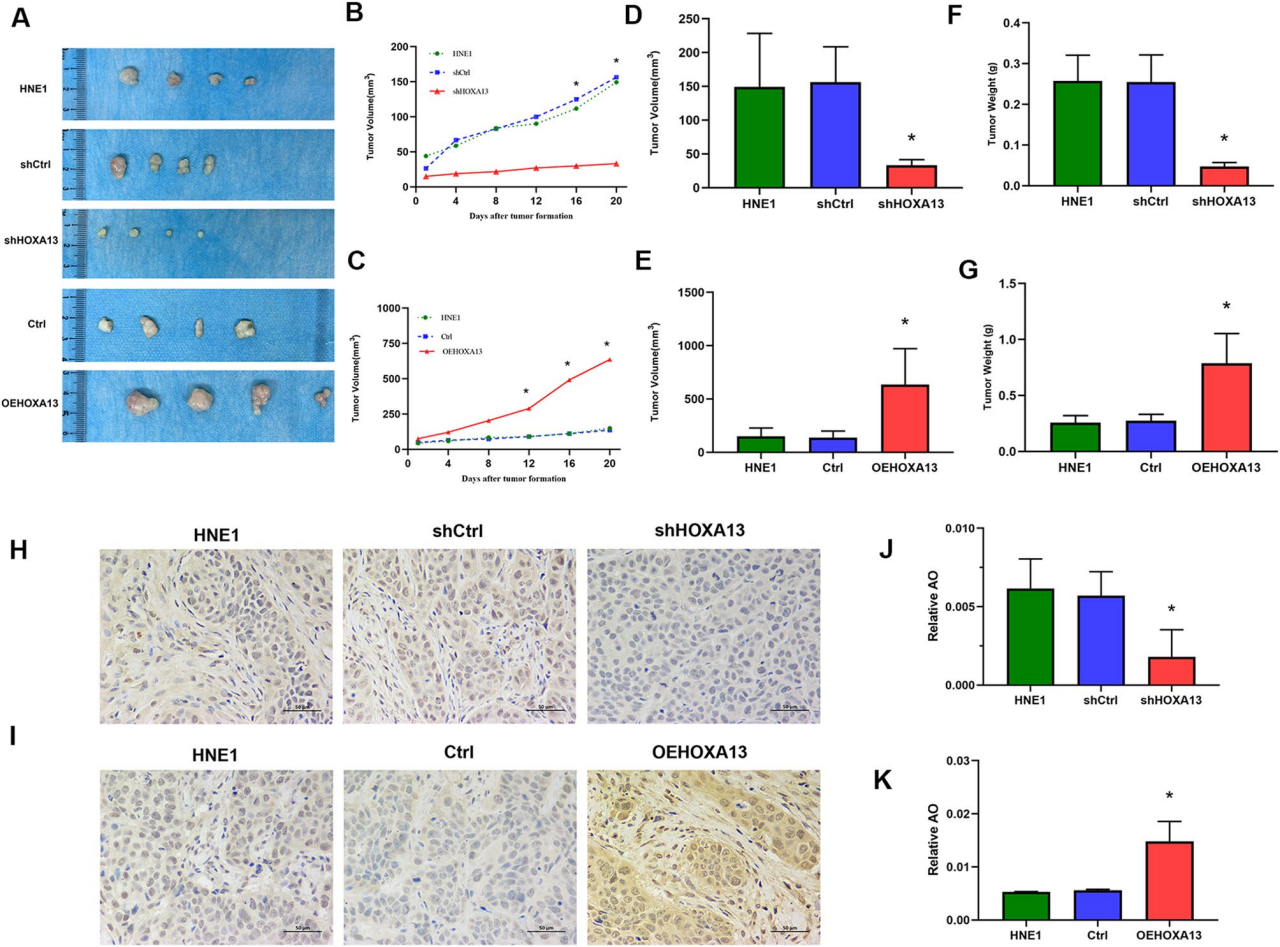
Silencing of HOXA13 inhibit the growth of HNE1 cell xenograft tumors. (**A**) Xenograft tumors formation in nude mice. (**B**–**G**) The volume, and mass of xenograft tumors in each group. (**H**–**I**) Immunohistochemical detection of HOXA13 protein expression in xenograft tumor tissues. (**J**–**K**) OA values measured by immunohistochemical images in each group. (*, *P* < 0.05).

The results of the xenograft tumour model showed that all nude mice grew tumourus. After silencing HOXA13, the volume and mass of xenograft tumourus were significantly decreased compared with those of the control group (*P* < 0.05). However, the opposite results were obtained after the overexpression of HOXA13 (*P* < 0.05) (Fig. 5A–G) Immunohistochemistry showed that after silencing HOXA13, the expression of HOXA13 protein was decreased (*P* < 0.001), while the expression of HOXA13 protein was increased after the overexpression of HOXA13 (*P* < 0.001) (Fig. 5H–K).

### HOXA13 regulates the expression of Snail and MMP-2 in HEN1 cells and NPC tissues

**Figure 6:**
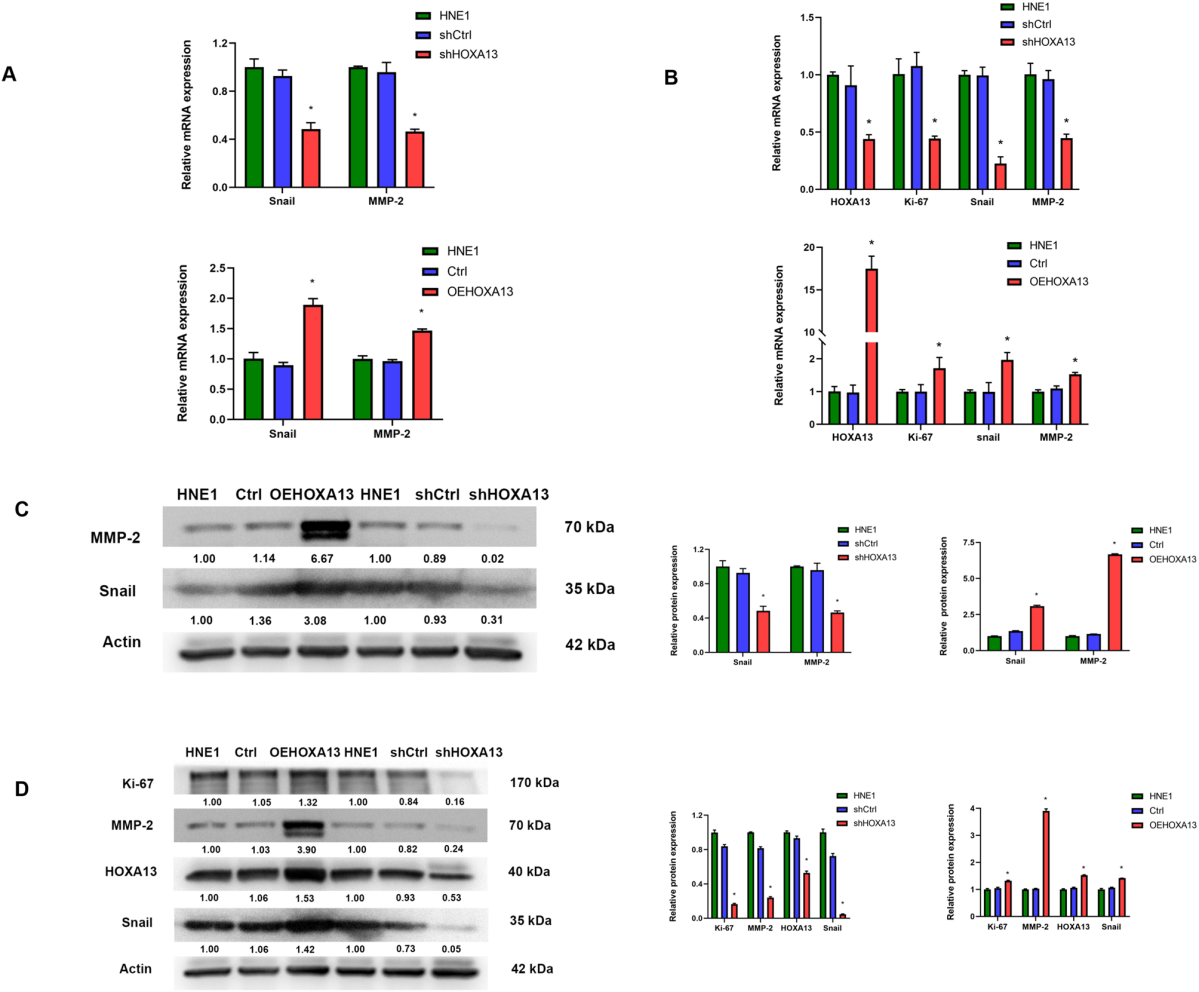
HOXA13 regulates the expression of Snail and MMP-2. (**A**) qRT-PCR assay detects the mRNA expression of Snail and MMP-2 in HNE1 cells. (**B**) qRT-PCR assay detects the mRNA expression of HOXA13, Snail, MMP-2 and Ki-67 in xenograft tumorus. (**C**) Western blotting assay detects the protein expression of Snail and MMP-2 in HNE1 cells. (**D**) Western blotting assay detects the protein expression of HOXA13, Snail, MMP-2 and Ki-67 in xenograft tumors. The samples are derived from the same experiment and that blots were processed in parallel. Numbers represent ratios of gray value of target band to gray value of Actin. (*, *P* < 0.05).

**Figure 7:**
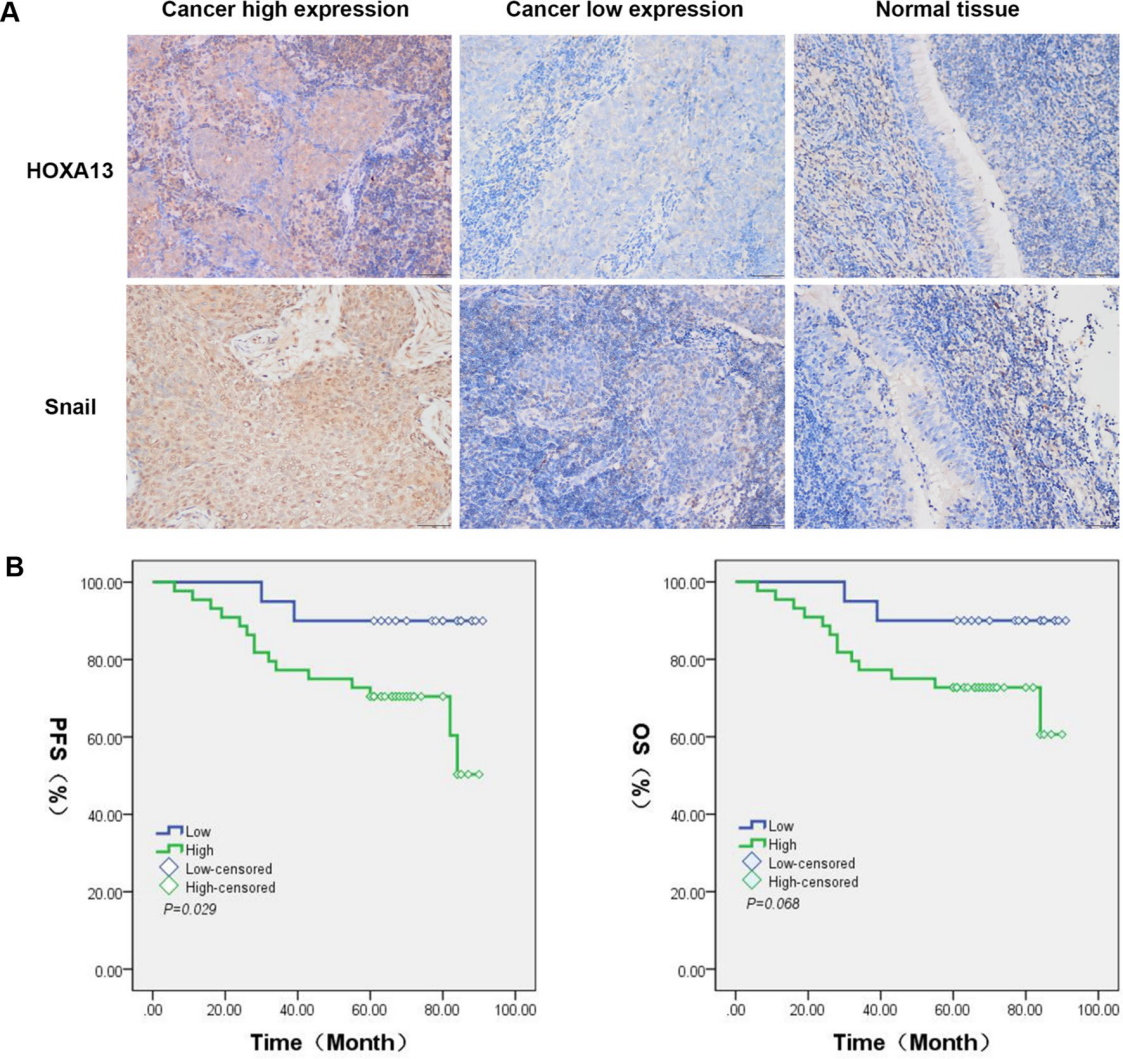
The progression of HOXA13 in NPC. (**A**) Representative immunohistochemistry staining of HOXA13 and Snail in nasopharyngeal carcinoma tissue and normal tissue. Scale bars, 200 μm. (**B**) Kaplan–Meier survival curves for low or high HOXA13 expression. PFS, Progression-free survival rate; OS, Overall survival rate.

Snail and MMP-2 are important markers in the process of cancer invasion. We used qRT-PCR and Western blotting to explore the role of Snail and MMP-2 in NPC HNE1 cells. The results showed that silencing of HOXA13 decreased Snail and MMP-2 mRNA and protein levels (*P* < 0.05), while overexpression of HOXA13 increased Snail and MMP-2 mRNA and protein levels (*P* < 0.001) (Fig. 6A, C). To study whether HOXA13 can still regulate Snail and MMP-2 in vivo, qRT-PCR and Western blotting were performed concurrently on xenograft tumour tissues, and the results were consistent with the above results. In addition, we detected the mRNA and protein expression of Ki-67 in xenograft tumour tissues, and found that overexpression of HOXA13 increased the expression of Ki-67, while silencing induced the reverse effect (*P* < 0.05) (Fig. 6B, D). Further research showed that HOXA13 and Snail were highly expressed in nasopharyngeal carcinoma tissues (68.75% (44/64) and 62.50% (40/64), respectively), significantly higher than normal tissues (6.67% (2/30) and 13.33% (4/30)), respectively) (*P* < 0.05). Nasopharyngeal carcinoma patients with high expression of HOXA13 or Snail had worse stage N, and neither of them had significant correlation with distant metastasis (stage M). (Fig. 7A, Supplementary Table 1). Moreover, the progression-free survival rate of patients with high expression of HOXA13 was lower than that of patients with low expression of HOXA13, but there was no significant difference in overall survival between these two groups (Fig. 7B). Spearman bivariate correlation analysis method was used to analyze the relationship between HOXA13 and Snail expression in nasopharyngeal carcinoma samples, and the result was positive correlation (*r* = 0.661, *P* < 0.05) (Supplementary Table 2).

## Discussion

The dysregulation of HOXA13 expression is associated with cancers^14,19,30–32^. Patients with high expression of HOXA13 had higher histological grades and higher T/N stages^14^, A study revealed that HOXA13 plays a cancer-promoting role by directly downregulating DHRS2 and upregulating MDM2 and promotes drug resistance through the p53 pathway^33^. HOXA13 can also exhibit opposing oncogenic effects in the same type of tumour, depending on its cell characteristics. For example, in CS12 gastric cancer cells, HOXA13 behaves as a cancer-promoting gene, but after reprogramming CS12 cells into gastric cancer-induced pluripotent stem cell-like cells, the oncogenic effect of HOXA13 disappeared, and it exhibited tumour-suppressive properties^34^. In the present study, we found that HOXA13 was upregulated in NPC and promoted the proliferation, migration and invasion of NPC HNE1 cells.

The view that EMT plays a vital role in the migration and invasion of malignant tumorus have been widely accepted^35^. As important indicators of invasion, Snail and MMPs are often studied in cancer research. Snail can directly bind to the E-cadherin promoter in the E-box sequence to inhibit E-cadherin expression^36^. Ren et al.^37^ found that Snail can be used as a downstream target of HOPX in NPC. HOPX recruits HDAC to the Snail promoter and epigenetically inhibits Snail transcription, thereby inhibiting the invasion of NPC cells.

MMPs are a zinc-dependent endopeptidases family that can cleave all extracellular matrix substrates. During EMT, MMPs can interact with integrins or target transmembrane proteins to reduce intercellular adhesion^38^ and promote the adhesion of cells to the ECM through integrins to activate FAK^39^. Li et al., performed immunohistochemical analysis on samples from 144 NPC patients and found that MMP-2 was highly expressed in NPC patient samples, and its expression level was positively correlated with the T and M stages of tumors. After overexpression of MMP-2, the expression of N-cadherin, fibronectin, and slug increased, while the expression of E-cadherin decreased, and cell adhesion decreased, which promoted EMT and tumour invasion^40^. In addition, there was an interaction between Snail and MMP-2. After knocking down Snail, the activity of MMP-2 is greatly decreased, and the tumour size and metastasis rate of ovarian cancer were decreased, suggesting that Snail promotes the growth and metastasis of ovarian cancer by promoting MMP-2 activity^41^. In the present study, upon silencing HOXA13, the mRNA and protein expression levels of both Snail and MMP-2 were decreased both in vivo and in vitro, and overexpression of HOXA13 reversed this effect, suggesting that HOXA13 promotes the invasion of NPC by upregulating Snail and MMP-2. However, the downside of our study is that we did not explore the pathways through which Snail and MMP-2 regulate the progression of NPC in depth or whether there are interactions between Snail and MMP-2. These will be the focus of future studies by our research group.

In summary, HOXA13 plays a role as a cancer-promoting gene in NPC and can promote the proliferation, migration, and invasion of NPC HNE1 cells by upregulating Snail and MMP-2. We speculate that HOXA13 could become a target for the diagnosis and treatment of NPC.

## Materials and methods

### Data mining of biological information

The available datasets GSE 53819 (https://www.ncbi.nlm.nih.gov/geo/query/acc.cgi?acc=GSE53819) and GSE64634 (https://www.ncbi.nlm.nih.gov/geo/query/acc.cgi?acc=GSE64634) were downloaded from the GEO database. GSE53819 contains the gene expression profiles of 36 paired samples (18 NPC tissue samples and 18 normal nasopharyngeal tissue samples). GSE64634 contains the gene expression profiles of 16 unpaired samples (12 NPC tissue samples and 4 normal nasopharyngeal tissue samples). The limma software package in R was used to normalize and annotate the original data of the two datasets. Differentially expressed genes (DEGs) were screened using *P* < 0.05 and fold change > 2 (i.e., |log_2_-fold change|> 1) as the threshold. The pheatmap package in R was used to generate a heatmap and volcano maps of the DEGs, and then the expression values of HOXA13 in each tissue sample were analyzed.

### Cell lines, cell culture and tissue samples

Human HNE1 NPC cells were provided by the laboratory of West China Hospital of Sichuan University and were preserved at the Department of Otorhinolaryngology, Head and Neck Surgery, Affiliated Hospital of Southwest Medical University. Cells were cultured routinely in complete medium containing 10% FBS and 1% penicillin–streptomycin antibiotics at 37 °C in a 5% CO_2_ saturated humidity incubator. 64 nasopharyngeal carcinoma paraffin-embedded tissues and 30 normal tissues were collected from the Affiliated Hospital of Southwest Medical University. The study was approved by the Clinical Trial Ethics Committee of the Affiliated Hospital of Southwest Medical University.

### Viral vector construction and cell transfection

The design of the HOXA13 interference fragment, vector construction, and virus packaging process were completed by Landm Biotech (Guangzhou, China). The HOXA13 (5′ACGAGCTGTACAAGATGACAGCCTCCGTGCTCCTCCACCC 3′) sequence or the negative control sequence for the overexpression vector was cloned into the lentiviral vector pLVX-IRES-EGFP-NEO. HOXA13-shRNA (5′ TCGCGGACAAGTACATGGATACTCGAGTATCCATGTACTTGTCCGCGATTTTT 3′) or the negative-control silencing empty vector was inserted into the lentiviral vector L202 (L202 CMV.CopGFP-2A-Puro.H1.shRNA). The recombinant plasmid, PMD2.G, and psPAX2 were then cotransfected into 293 T cells using Lipofectamine 2000 (Invitrogen, USA), and the viral supernatant was collected to infect HNE1 cells. All stably transfected cells were selected with 2 µg/ml puromycin (InvivoGen, USA). The expression of HOXA13 in each group of cells was verified by qRT-PCR and Western blotting. The cells in each group were named as follows: silencing experiment: HNE1 (untreated HNE1 cells), shCtrl (HNE1 cells containing empty silencing vector), shHOXA13 (HNE1 cells containing stable HOXA13 silencing vector); overexpression experiment: HNE1 (untreated HNE1 cells), Ctrl (HNE1 cells containing empty overexpression vector), and OEHOXA13 (HNE1 cells stably overexpressing HOXA13).

### MTT assay

MTT assays and plate colony formation assays were used to detect cell proliferation ability. The six groups of cells were diluted with complete culture medium to 1000 cells/ml cell suspension, and 200 μl of cell suspension was added to each well of a 96-well plate, with three replicate wells and blank wells for each group. The cells were cultured in an incubator. On days 1, 2, 3, and 4, 20 μl of MTT solution (Solarbio, China) was added to each well. The culture medium was discarded after conventional culture for 4 h, and 150 μl of dimethyl sulfoxide solution (Beyotime, China) was added to each well. The plates were shaken at low speed for 10 min until the crystals at the bottom had fully dissolved. The optical density (OD) value of each well was measured by a microplate reader (Thermo Fisher Scientific, USA) at a wavelength of 490 nm.

### Plate colony formation assay

Cells were seeded in six-well culture plates (200 cells per well) and cultured for 7–14 days. The culture was terminated when more cell clusters (composed of 50 or more cells) appeared under the microscope. Cells were fixed in 4% paraformaldehyde (Solarbio, China) for 30 min, stained with crystalline violet (Servicebio, China) for 30 min, washed, and air-dried. The clone formation rate was calculated as follows: colony formation rate = (total number of clones per well/number of cells inoculated per well) × 100%. Using the unified standard of colony counting, a cell cluster composed of 50 or more cells was counted as one clone.

### Transwell assay

A total of 1 × 10^5^ cells were inoculated into the upper chamber of a 24-well transwell chamber coated with (invasion) or without (migration) Matrigel (BD, USA), and 600 μl of complete medium containing 10% FBS was added to the lower chamber. After incubation at 37 °C for 24 h, the cells that invaded or migrated to the chamber membrane were fixed with 4% paraformaldehyde for 30 min, washed with PBS, and fixed with 0.1% crystal violet staining solution.

### Quantitative real-time polymerase chain reaction (qRT-PCR)

RNA was extracted from tissues or cells using RNAiso Plus (Takara, Japan) according to the manufacturer’s instructions. The RNA concentration was detected using a NanoDrop 2000 (Thermo Fisher Scientific, USA). cDNA was synthesized using EasyScript All-in-One First-Strand cDNA (TransGen, China). qRT-qPCR experiments were performed using TransStart Tip Green qPCR SuperMix (+ Dye II) (TransGen, China). The reaction program was as follows: 50 °C for 2 min; 95 °C for 2 min; and 40 cycles of 95 °C for 15 s and 60 °C for 32 s. The relative gene expression level was calculated using the formula 2^−△△Ct^. The primers used (Sangon, China) are listed in Table 1.

**Table 1:** qRT-PCR primers used in the present study.

Genes	Forward primer (5′–3′)	Reverse primer (5′–3′)
HOXA13-human	GCTGGAACGGCCAAATGTACT	TTGGTATAAGGCACGCGCTTC
Snail-human	TCTGCGGAACCTGCGGGAAG	TGCGACCACAACCAACTACGATG
MMP-2-human	TGGCACCACCGAGGACTATGAC	ACATGGGGCACCTTCTGAATTTCC
GAPDH-human	GGGAAACTGTGGCGTGAT	GAGTGGGTGTCGCTGTTGA
β-actin-human	CATCCTGCGTCTGGACCTGG	TAATGTCACGCACGATTTCC
HOXA13-mouse	ATGACAGCCTCCGTGCTCCTC	GCCGCCCCTTCCATGTTCTTG
Snail-mouse	TCACCTTCCAGCAGCCCTACG	CCAGGAGAGAGTCCCAGATGAGG
MMP-2-mouse	TGGCACCACCGAGGACTATGAC	ACATGGGGCACCTTCTGAATTTCC
β-actin-mouse	GGCTGTATTCCCCTCCATCG	CCAGTTGGTAACAATGCCATGT

### Western blotting assay

The tissues and cells were collected, added to radioimmunoprecipitation assay lysis buffer (UBI, China), and fully lysed on ice for 30 min. The protein content of each group was detected using a bicinchoninic acid protein concentration assay kit (UBI, Shanghai China). Equal amounts of protein samples (20 μg) were subjected to sodium dodecyl sulfate–polyacrylamide gel electrophoresis (Beyotime, China), transferred to poly (vinylidene fluoride) membranes (Millipore, USA), blocked at room temperature for 2 h with 5% skim milk (WHIGA, China), incubated overnight at 4 °C with primary antibodies, and then incubated with secondary antibodies (anti-rabbit or anti-mouse, 1:5000, Abcam, USA) containing horseradish peroxidase (HRP) for 2 h at room temperature. Finally, cut the membranes after hybridisation with antibodies and developed with ECLPLUS luminescent solution (Clinx, China). The primary antibodies were as follows: HOXA13 (1:1000, Abcam, USA), Snail (1:1000, ABclonal, USA), MMP-2(1:1000, ABclonal, USA), Ki-67 (1:1000, Abcam, USA), Actin (1:1000, Abcam, USA).

### Xenograft tumour model

The animal experiment was approved by the Experimental Animal Ethics Committee of Southwest Medical University (NO. 2020912), and we confirm that all experiments were performed in accordance with relevant named guidelines and regulations complied with the ARRIVE guidelines. 5-week-old female BALB/c nude mice were obtained from SPF (Beijing) Biotechnology Co., Ltd., China, and were housed in the specific pathogen-free (SPF) animal rooms of the Department of Oncology, Affiliated Hospital of Southwest Medical University. Nude mice were randomly divided into five groups (N = 4). Then, 100 μl of 1 × 10^7^ HNE1, shCtrl, shHOXA13, Ctrl or OEHOXA13 cells in the logarithmic phase of growth were injected into the subcutaneous tissues next to the left thigh of the nude mice. When the tumour was visible to the naked eye, its volume was measured every four days using a Vernier caliper. The volume was calculated using the following formula: volume = 0.5 × length × width^2^. On the 21st day after tumour formation, all nude mice were sacrificed by cervical dislocation. The xenograft tumours were dissected and weighed. The tumour tissues were collected for subsequent immunohistochemical detection, qRT-PCR, and Western blotting.

### Immunohistochemical analysis

The expression of HOXA13 and Snail in tissues was detected by immunohistochemistry. The 4% paraformaldehyde-fixed and paraffin-embedded xenograft tissue was cut into 3 mm thick sections, after dewaxing and hydration, the tissue sections were placed in a retrieval box filled with citric acid antigen retrieval buffer (pH 6.0, Servicebio, China) for antigen retrieval in a microwave. After natural cooling, the sections were washed with PBS (pH 7.4, HyClone, USA) 3 × for 5 min. The sections were incubated in 3% hydrogen peroxide solution (Sinopharm Chemical Reagent Co., Ltd., China) at room temperature in the dark for 25 min and then washed with PBS 3 times for 5 min. The tissues were evenly covered with 3% BSA (BioFroxx, Germany) and blocked at room temperature for 30 min. The tissues were incubated with the primary antibody HOXA13 (1:200; Abcam, USA) overnight at 4 °C in a wet box. After washing with PBS three times, goat anti-rabbit (1:200, Servicebio, China) was added and incubated at room temperature for 50 min. After three more washes, 3,3-diaminobenzidine (DAB) color development solution (DAKO, Denmark) was added. The slides were counterstained with hematoxylin, dehydrated, sealed, microscopically observed, and photographed. The immunohistochemistry results were analyzed in Image-Pro Plus 6.0, in which we applied the same brown-yellow color as the uniform standard for judging whether images were positive. The cumulative optical density (IOD) and the pixel area of the tissue (AREA) in each positive photo were analyzed, and the average optical density (AO value) was calculated by AO = IOD/AREA. The higher the AO value, the higher the expression level of the positive protein.

### Statistical analysis

SPSS 17.0 (SPSS, USA) were used for data analysis. Two groups were compared by Student’s *t* test. Multiple groups were compared by analysis of variance. All analyses were performed using the two-tailed method. The data are expressed as the mean ± standard deviation (SD) of three experiments. The overall survival (OS) and Progression-free survival rate (PFS) of the groups were compared using the Kaplan–Meier method. The correlation of HOXA13 and Snail in NPC patients was analyzed by Spearman bivariate correlation analysis. *α* = 0.05 (*P* < 0.05) was the test level for significance.

### Ethical approval

The study was approved by the Experimental Animal Ethics Committee of Southwest Medical University (NO. 2020912), and we confirm that all experiments were performed in accordance with relevant named guidelines and regulations complied with the ARRIVE guidelines. Protocols involving human research participants had been performed in accordance with the Declaration of Helsinki and informed consent was obtained from all subjects.

### Supplementary Information

Supplementary Tables.

## Data Availability

All data generated or analysed during this study are included in this article.
