# Supplementary material for: HOXA13 promotes the proliferation, migration, and invasion of nasopharyngeal carcinoma HNE1 cells by upregulating the expression of Snail and MMP-2
Source: Sci Rep. 2023 Aug 10;13:12978. doi: 10.1038/s41598-023-40041-8 (PMC10415404; doi:10.1038/s41598-023-40041-8)
Supplement: Supplementary file 1 — Supplementary Tables. [file 41598_2023_40041_MOESM1_ESM.docx]

**HOXA13 promotes the proliferation, migration, and invasion of nasopharyngeal carcinoma HNE1 cells by upregulating the expression of Snail and MMP-2**

Jinping Liu, Huajun Feng, Dingting Wang, Yuanyuan Wang, Jian Luo, Shengen Xu, Feipeng Zhao, Gang Qin

Supplementary tables

Supplementary Table 1 Correlation of HOXA13 and Snail expression with clinicopathologic features in nasopharyngeal carcinoma patients.

| Characteristic | HOXA13 | | *χ^2^* | *P* | Snail | | *χ^2^* | *P* |
| --- | --- | --- | --- | --- | --- | --- | --- | --- |
|  | High (n  = 44) | Low (n = 20) |  |  | High (n  = 40) | Low (n = 24) |  |  |
| Gender |  |  |  |  |  |  |  |  |
| Male | 35 | 12 | 2.693 | 0.131 | 30 | 17 | 0.134 | 0.774 |
| Female | 9 | 8 |  |  | 10 | 7 |  |  |
| Age |  |  |  |  |  |  |  |  |
| ＞51 | 23 | 13 | 0.905 | 0.420 | 21 | 15 | 0.610 | 0.603 |
| ≤51 | 21 | 7 |  |  | 19 | 9 |  |  |
| Histological grade* |  |  |  |  |  |  |  |  |
| Ⅰ | 4 | 2 | 4.242 | 0.120 | 3 | 3 | 1.904 | 0.386 |
| Ⅱ | 25 | 6 |  |  | 22 | 9 |  |  |
| Ⅲ | 15 | 12 |  |  | 15 | 12 |  |  |
| T |  |  |  |  |  |  |  |  |
| T_1-2_ | 25 | 11 | 0.018 | 1.000 | 22 | 14 | 0.068 | 1.000 |
| T_3-4_ | 19 | 9 |  |  | 18 | 10 |  |  |
| N |  |  |  |  |  |  |  |  |
| N_0-1_ | 9 | 12 | 9.754 | 0.003 | 8 | 13 | 7.943 | 0.007 |
| N_2-3_ | 35 | 8 |  |  | 32 | 11 |  |  |
| M |  |  |  |  |  |  |  |  |
| M_0_ | 44 | 18 | 4.542 | 0.094 | 40 | 22 | 3.441 | 0.137 |
| M_1_ | 0 | 2 |  |  | 0 | 2 |  |  |
| Clinical stage** |  |  |  |  |  |  |  |  |
| Early stage | 4 | 7 | 6.485 | 0.027 | 3 | 8 | 7.033 | 0.014 |
| Advanced stage | 40 | 13 |  |  | 37 | 16 |  |  |

* Histological grade Ⅰ: Keratinizing squamous cell carcinoma, Ⅱ: Nonkeratinized differentiated carcinoma, Ⅲ: Nonkeratinized differentiated carcinoma; ** Clinical stage: Early stage: TNM Ⅰ-Ⅱ，Advanced stage : TNM Ⅲ-Ⅳ.

Supplementary Table 2 The correlation between HOXA13 and Snail in nasopharyngeal carcinoma tissues using Spearman test.

|  |  | Snail | | r | *P* |
| --- | --- | --- | --- | --- | --- |
|  |  | High expression | Low expression |  |  |
| HOXA13 | High expression | 37 | 7 | 0.661 | 0.000 |
|  | Low expression | 3 | 17 |  |  |
